# Personal profile of medical students selected through a knowledge-based exam only: are we missing suitable students?

**DOI:** 10.3402/meo.v21.29705

**Published:** 2016-04-12

**Authors:** Milena Abbiati, Anne Baroffio, Margaret W. Gerbase

**Affiliations:** Faculty of Medicine, Unit of Development and Research in Medical Education, University of Geneva, Geneva, Switzerland

**Keywords:** selection, profile, medical students, undergraduate, personal characteristics, assessment

## Abstract

**Introduction:**

A consistent body of literature highlights the importance of a broader approach to select medical school candidates both assessing cognitive capacity and individual characteristics. However, selection in a great number of medical schools worldwide is still based on knowledge exams, a procedure that might neglect students with needed personal characteristics for future medical practice. We investigated whether the personal profile of students selected through a knowledge-based exam differed from those not selected.

**Methods:**

Students applying for medical school (*N*=311) completed questionnaires assessing motivations for becoming a doctor, learning approaches, personality traits, empathy, and coping styles. Selection was based on the results of MCQ tests. Principal component analysis was used to draw a profile of the students. Differences between selected and non-selected students were examined by Multivariate ANOVAs, and their impact on selection by logistic regression analysis.

**Results:**

Students demonstrating a profile of diligence with higher conscientiousness, deep learning approach, and task-focused coping were more frequently selected (*p*=0.01). Other personal characteristics such as motivation, sociability, and empathy did not significantly differ, comparing selected and non-selected students.

**Conclusion:**

Selection through a knowledge-based exam privileged diligent students. It did neither advantage nor preclude candidates with a more humane profile.

The art of medicine and the role of medical doctors are constantly evolving as a response to the changing healthcare needs of society. As stated in the CanMeds framework (2005), ‘the Role of Medical Expert is central to the function of physicians and draws on the competencies included in the Roles of Communicator, Collaborator, Manager, Health Advocate, Scholar and Professional’ (1). This is why organizations such as the Association of American Medical Colleges and the Royal College of Canada have tried to establish what constitutes a ‘good doctor’ and hence define the core competencies that future medical graduates should possess (e.g., academic excellence, inter- and intrapersonal skills) in order to become qualified and caring professionals (2–4). Beyond academic performance, there is the current debate on which personal competencies are important for students entering medical school, and which reliable and valid methods to apply for selecting those students with the greatest potential to become efficient, professional, and caring future doctors (5, 6).

There is a general agreement about the academic competencies necessary for students to succeed during medical school. A consistent body of evidence is currently available to show that cognitive measures of high school performance such as the grade point average (GPA) score or the scores on medical school aptitude tests predict academic success during medical school (7–10). However, less clear are the personal characteristics required to enter medical school and the methods to assess these features which would be suitable for inclusion in the selection process (11). Previous work has shown that personality traits, coping with stress, motivation, and approach to learning are characteristics that seem to partially predict future academic and professional performance (12). Furthermore, recent studies in medical education recommend that personality measures should be included not only to assess in-training doctors but also in the admission criteria for medical school entrance (13).

To date, a considerable number of universities from different countries have adopted admission policies, most of which include cognitive measures, such as the high school GPA score, and scores on aptitude tests such as the Medical College Admission Test (MCAT) (14). In addition, some medical schools mainly in the United States, Canada, and Belgium have added ways to evaluate personal characteristics of medical school candidates, by means of personal statements, letters of recommendation, adhesion to extracurricular activities, interviews, psychological tests, multiple mini-interviews, and situational judgment tests (7). However, as pointed out by Powis in a recent review, good selection of medical students is ‘an unresolved challenge’ and some medical schools still do not have admission policies (15). In addition, a great number of medical schools worldwide still apply knowledge-based exams as the only procedure to select candidates for medical studies. The question then arises on the personal characteristics of students selected through a single cognitive procedure, and whether students with a more humane personal profile would be precluded by this process. The approach to this question needs to take into account gender as a personal characteristic. As shown before, women present different personality characteristics compared with men, the former being more agreeable and emotional (16), more empathetic (17), and using different strategies when coping with stress (18).

At our institution, students of the medical school are selected at the end of the first year of university studies on the basis of performance in biannual knowledge exams. Therefore, we took the opportunity of having students in their ‘natural’ setting of studies, to investigate whether this selection process might disfavor students with suitable individual features to become caring doctors in the future. Hence, this study aimed at 1) assessing the personal profile of students entering the first year of medical school at the University of Geneva, by using a set of questionnaires; and 2) investigating differences in profile between selected and non-selected students, further examining whether these differences would impact selection for medical studies. The role of gender was also considered.

## Methods

This study belongs to a larger research project designed to follow a cohort of medical students throughout the whole curriculum (6 years), by assessing how their cognitive and personal characteristics and their educational context impact academic performance and career intentions over time.

### Participants

Eligible participants were all students officially enrolled in the first year of medical school during the academic year 2011–2012. Students received an email 10 days before the survey to inform them about the research project's main goals, the questionnaires’ content and the testing conditions (confidential****, voluntary participation). All students present in the classroom on the survey day received this information once again. Students who agreed to participate signed a written consent form, as appropriate. In order to match each student's data collected throughout the duration of the study, they reported their student ID. Confidentiality was however ensured, since researchers had no access to the codes identifying students. A technical administrator not directly linked to the study was responsible for keeping the students’ codes and identities. The Chair of the Ethics Committee for Public Health Research designated our study as exempted from formal review.

### Selection process for medical school

Selected students were students admitted to the second year of medical studies, that is, those having passed two biannual written exams during the first year of medical studies. Both written exams consisted of 120 multiple-choice questions testing the knowledge acquired during the first-year lectures (e.g., physics, chemistry, anatomy, physiology, and genetics). A permanent board of basic and medical science experts is entitled to conceive and review the exam's content, as well as to regularly update the exam with new questions. In order to pass, students have to get a minimum grade of 4 out of a maximum of 6 in both examinations.

As a period of 2 academic years is allowed to complete the first study-year, the student population (about 450) consisted of a mixture of freshmen (about two-thirds of the students, having started in the academic year 2011–2012) and repeaters (about one-third of the students, having started in the academic year 2010–2011), from whom about 160 students were selected to pursue medical school from the second to the sixth year of studies.

### Data

Data used in the study were derived from former students’ records kept by the medical school and from prospective students’ answers to a survey.

#### Former students’ records

Students’ aptitude for medical studies was estimated from their scores on the EMS (Eignungstest für das Medizinstudium in der Schweiz or Aptitude test for medical studies in Switzerland) (19). The EMS is the medical studies admission test used in the German-speaking part of Switzerland. The test was administered in Geneva as a compulsory but not selective screening test from 2010 to 2012 (20). Like the US MCAT, the EMS assesses the capacity for reasoning and solving problems, as well as the ability to generalize and transfer knowledge. However, it does not assess basic concepts in biology, chemistry, or physics, or communication skills or social competencies. Test scores range from 0 (low) to 100 (high) as a percentage rank.

#### Prospective survey

The survey was taken by students in May 2012. It included several questionnaires assessing students’ personal characteristics.

Sociodemographics incorporated data on age, gender, nationality (Swiss, European or other), and type of high school diploma (scientific vs. other). As an indirect measure of a student's socioeconomic level, parents’ current occupations were asked for and categorized as: elementary (e.g., worker, artisan), professionals (e.g., lawyer, physician), clerks (e.g., secretary), managers (e.g., chief executive officer), and other (e.g., housewife/man) (21). Information on the parents’ highest educational achievement was gathered, that is, primary, lower secondary, upper secondary, tertiary, and other (22).

Motivations for becoming a doctor: students were invited to indicate on a 6-point categorical scale (1=‘not important at all’ to 6=‘very important’) the importance they accorded for the following 10 motivations for becoming a doctor: vocation, mission, altruism, reward, prestige, academic activity, private practice, treat illness, care for patients, and save lives. The 10 motivations used in this study were mostly derived from terms found in descriptions of the self-determination theory (23), and this construct has not been validated. In order to test its reliability, we performed a factor analysis by aggregating the 10 motivations using a principal component analysis (PCA) with varimax rotation. It yielded three main components, which met the criterion for selecting factors (eigenvalues larger than 1) and explained 65% of total variance (KMO *p*=0.756, Bartlett *p*<0.001). The motivations loading on the three components allowed us to label each of them as a motivation type: intrinsic (vocation, mission, and altruism), extrinsic (reward, prestige, academic activity, and private practice), and care (patient, illness, and lives).

Learning approaches were measured with the Revised two-factor Study Process Questionnaire (R2-SPQ) (24), consisting of 20 items scored on a 5-point Likert scale (1=‘this item is never or only rarely true of me’ to 5=‘this item is always or almost always true of me’). R2-SPQ evaluates two major non-exclusive learning approaches: the Deep Approach (SPQ-DA) and the Surface Approach (SPQ-SA). The total scores of the SPQ-DA and the SPQ-SA range from 10 (rare) to 50 (always). Conceptually, a learner with a high SPQ-DA tries to understand what he or she is studying, and to relate ideas to previous knowledge and experience. A learner with a high SPQ-SA score memorizes facts and figures in order to pass the exam, and accomplishes what is required with the minimum effort. The R2-SPQ instrument measuring two learning approaches was developed by Biggs et al. (24) and has been recently re-validated by Socha et al. (25). As it was not available in French, the previously validated English items were translated into French and back-translated into English by two independent reviewers for quality control. The calculated Cronbach's coefficients of the translated scales were 0.77 for SPQ-DA and 0.74 for SPQ-SA.

Coping styles were assessed by the Coping Inventory for Stressful Situations (CISS) (26), which consists of 48 items rated on a 5-point Likert scale (1=‘not at all’ to 5=‘a lot’, maximum score=80). CISS assesses three main coping strategies that individuals might use when facing stressful situations, that is, reacting emotionally (emotional coping), focusing on the task (task coping), or by denial and running away (avoidant coping). This questionnaire is widely used in the domain of psychology and has been validated in a number of languages including French.

Personality traits were measured with the NEO Five Factor Inventory (NEO-FFI) (27) which is the short 60-item version of the classic Revised NEO Personality Inventory (NEO PI-R). NEO PI-R is the assessment instrument more often used to measure personality with the ‘Big Five’ model. This model assumes five underlying personality dimensions: neuroticism (N), extraversion (E), openness (O), conscientiousness (C), and agreeableness (A). NEO-FFI includes 12 items for each dimension scored on a 5-point Likert scale (0=‘strongly disagree’ to 4=‘strongly agree’, maximum score=48). High neuroticism matches with anxiety, moodiness, and sometimes loneliness and distress. High extraversion relates to being warm, affectionate, and enjoying dealing with others. High openness matches with curiosity and flexibility. High agreeableness relates to being sincere, showing full trust in others, and sometimes fearing to deceive others. High conscientiousness relates to efficiency and rationality, as well as to punctuality and organization. This questionnaire has been validated in different languages including French; standard norms for different populations have been determined (28).

Empathy was examined using the student's version of the Jefferson Scale of Empathy (JSE-S) (29), consisting of a 20-item questionnaire assessing students’ perception of the importance of empathy in the doctor–patient relationship. In the questionnaire, answers to questions are structured on a 7-point Likert scale (1=‘strongly disagree’ to 7=‘strongly agree’, maximum score=140. High scores indicate a strong tendency to value empathy in patients’ care. The Jefferson Scale of Physician Empathy (JSPE) has been validated in its original English version (29–31). A French translation of the JSPE is available (32), but the student's version of the JSPE in French has not been validated yet. For its use in this study, we slightly modified the wording of the French version of the JSPE to adapt it to students, respecting the original JSE-S version in English and performing a back translation. This version has been validated by our group showing similar psychometric results compared with the JSPE (personal communication).

### Statistical analysis

#### Students’ profiles

Results were expressed as percentages according to the overall population of students. Individual students’ total scores were calculated for 15 measures, that is, the three motivation types (intrinsic, extrinsic, and care, calculated as mean scores of the original motivations); the two learning approaches (SPQ-DA and SA); the three stress coping strategies (CISS task, emotion, avoidance); the five personality factors (NEO neuroticism, extraversion, openness, agreeableness, and conscientiousness); the empathy score (JSE-S), and the aptitude for medical studies (EMS expressed as percentage rank).

The students’ profiles were then derived from the 15 measures, using PCA with varimax rotation. It yielded six main components, with eigenvalues larger than 1 and explaining 66% of total variance (KMO *p*=0.63, Bartlett *p*<0.001). Each component obtained from the PCA procedure was defined as a facet that was labeled according to the measures loading on the component. Finally, each student was characterized according to these six new facets whose coordinates ranged from −1 to 1 (regression variables of each factor).

#### Differences in profile between selected and non-selected students

Two-factor multivariate ANOVA was run to analyze whether the six facets differed between students selected or not (‘selection’ and ‘gender’ used as factors). In addition, one-factor multivariate ANOVA was used to investigate whether, among selected students, freshmen and repeaters displayed different profiles.

#### Impact of differences in profile on selection for medical studies

Logistic regression analyses (odds ratio and 95% confidence intervals) were performed to examine facets predicting selection using a binary variable (selected vs. non-selected student).

A statistical significance level of *p*≤0.05 was adopted for all analyses unless otherwise specified.

## Results

### Medical students’ population

**Fig. 1:**
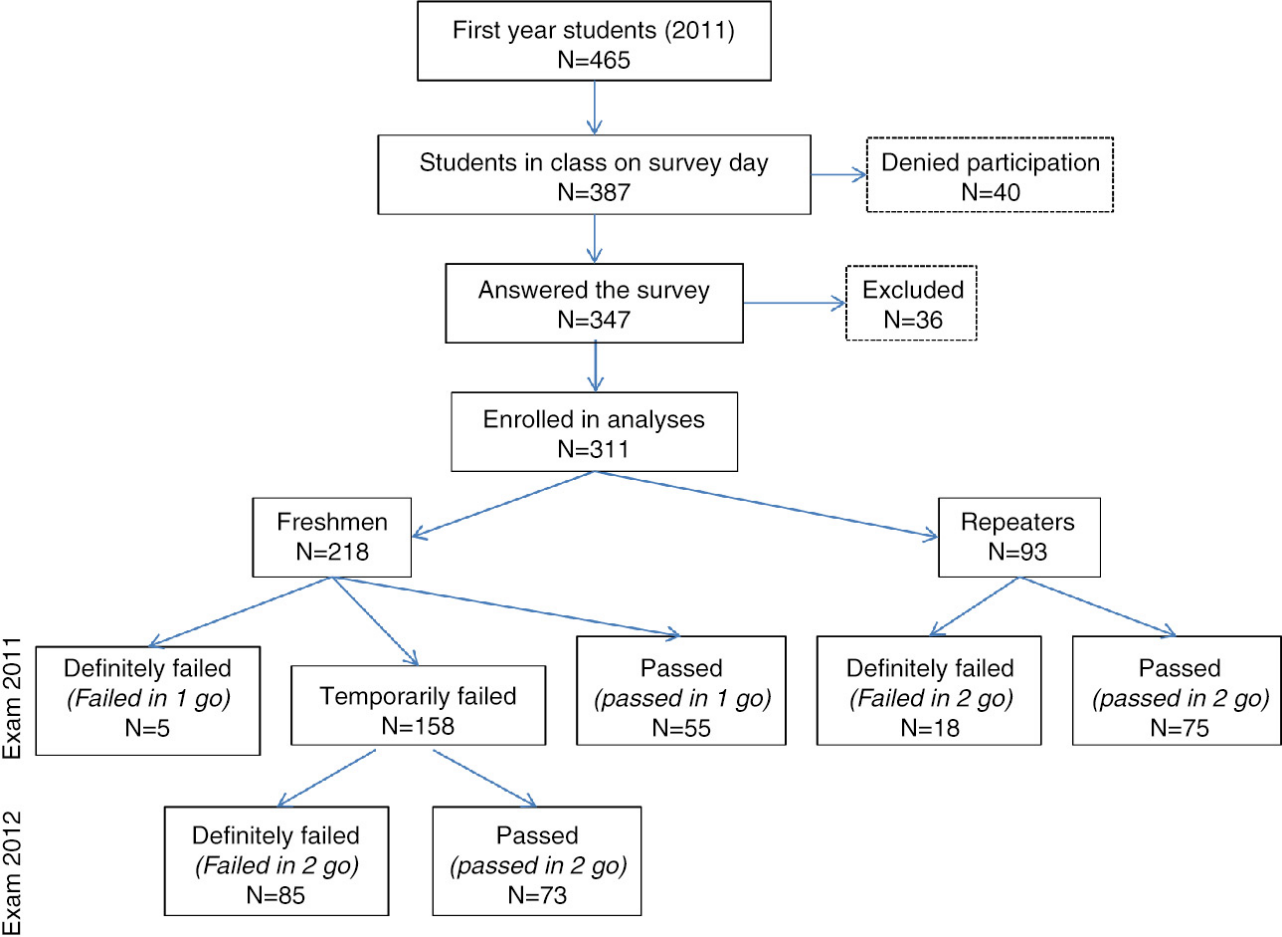
Selection and distribution of students participating in the study and follow-up of students over the 2-year selection process.

Figure 1 shows the distribution of students at the moment of enrollment in the study, while entering the first year of studies, and their status during the 2-year follow-up period of this study. On the survey day, out of 466 students enrolled in first year, 387 students (83%) attended class; among them, 347 (75%) students agreed to participate in the survey and 311 (67%) were eventually enrolled in analyses. Thirty-six students were excluded from analyses because of missing data (11 students failed to complete the identity code number and 25 students abandoned the classes before the exam sessions), which prevented a match of their questionnaire answers with the EMS and exam scores. From the 311 students eligible for analysis, 198 (64%) were women and 218 (70%) were freshmen. Among freshmen, 25% (*N=*55) passed ‘in one go’ after their first exam session (2011–2012) and 33% (*N*=73) passed ‘in two goes’ after their second exam session (2012–2013). Among repeaters, 80% (*N=*75) passed ‘in two goes’ after their second exam session (2011–2012). Hence, from our population of 311 student participants, 203 (65%) were finally selected for medical studies after the 2-year follow-up period. Out of 198 women, 118 (60%) were selected for medical studies. Out of 113 men, 85 (77%) were selected. The difference in the rates of selection between women and men was statistically significant (chi-square, 7.75; *p*=0.005).

As shown in Table 1, the majority of students enrolled in the study were women, aged 20 years, and Swiss citizens. Most students obtained a high school degree in science, but significantly fewer women than men (chi-square, 8.18; *p*=0.003). Nearly 65% of students’ parents had higher education and 50% of them worked as professionals or managers.

**Table 1:** Sociodemographic characteristics of students enrolled in the study, stratified by gender

		All (*N*=311)	Male (*n*=113)	Female (*n*=198)
Gender (%)		100	36.7	63.3
Age, median (range)		21 (15–38)	21 (18–32)	20 (15–38)
Nationality (%)	Swiss	81.4	80.2	82.6
	European	17.1	17.9	16.3
	Other	1.5	1.9	1.1
High school type (%)	Scientific	77	83.2	70.8*
	Other	23	16.8	29.2
Parents’ education (%)	Primary	7.5	5.8	9.2
	Secondary	22.5	22.3	22.6
	Tertiary	62.9	64.2	61.3
	Other	6.6	6.7	6.1
Parents’ occupations (%)	Elementary	5.4	4.9	5.9
	Clerks	15.4	12.4	18.3
	Professionals	41.8	45.8	37.8
	Managers	7.6	7.3	7.8
	Others	29.7	29.6	29.8

* Chi-square, *p*<0.05.

Table 2 displays the assessed students’ characteristics. Stratification by gender showed that women were significantly more emotional and empathetic than men; moreover, women presented stress coping (avoidance) and personal characteristics (neuroticism, agreeableness and conscientiousness) which significantly differed from men.

**Table 2:** Students characteristics, stratified by gender

		All	Males	Females	*p*
		*N*=311	*N*=113	*N*=198	Gender
Aptitude for medical studies		49.0 [23–83]	52.0 [25–83]	48.0 [23–73]	0.005*
Motivation	Care	6.0 [1–6]	5.7 [1–6]	6.0 [1–6]	0.060
	Intrinsic	5.0 [1–6]	5.0 [1–6]	5.0 [1–6]	0.567
	Extrinsic	4.0 [1–6]	4.0 [1–6]	4.0 [1–6]	0.533
Learning approaches	Deep	32.0 [12–49]	32.0 [16–45]	32.0 [12–49]	0.614
	Surface	23.0 [10–47]	23.0 [12–42]	23.0 [10–47]	0.354
Stress coping	Task	61.0 [39–80]	62.0 [43–76]	60.0 [39–80]	0.341
	Emotional	45.0 [16–73]	39.0 [16–65]	47.0 [21–73]	0.000*
	Avoidant	42.0 [15–72]	39.0 [15–60]	44.0 [19–72]	0.002*
Personality	Neuroticism	22.0 [1–46]	17.0 [1–35]	25.0 [2–46]	0.000*
	Extraversion	32.0 [16–44]	32.0 [17–42]	32.0 [16–44]	0.511
	Openness	29.5 [12–41]	30.0 [15–41]	29.0 [12–41]	0.594
	Agreeableness	30.0 [17–41]	29.0 [17–40]	30.0 [18–41]	0.046*
	Conscientiousness	34.0 [15–48]	33.5 [16–47]	35.0 [15–48]	0.014*
Empathy		114.0 [76–136]	110.0 [88–132]	115.2 [76–136]	0.000*

Median [Range].

* ANOVA by gender, *p*<0.05.

### Medical students’ personal profiles

The PCA, shown in Table 3, yielded six factors with the first three factors accounting for 44% of the variance. Each factor was then interpreted and labeled according to the co-varying characteristics. Factor 1 (19% of the variance), combining an approach to learning favoring deep over surface strategies, conscientiousness, and task coping, was named ‘diligent’. Factor 2 (14% of the variance), combining neuroticism and emotional coping, was named ‘emotional’. Factor 3 (11% of the variance), combining intrinsic and care motivations, was named ‘self-determined’. Factor 4 (8% of the variance), combining agreeableness and extraversion, was named ‘sociable’. Empathy, which did not clearly correlate with a single factor, but weakly with three different factors, was included in factor 4. Factor 5 (7% of the variance), combining aptitude for medical studies and openness, was named ‘intellectually flexible’. Factor 6 (7% of the variance), combining extrinsic motivation and avoidant coping, was named ‘externally driven’.

**Table 3:** Students’ profiles identified by principal factor analysis

	Measures	Higher component loadings	% variance explained	Facets
Factor 1	Deep approach	0.798	19	Diligent
	Conscientiousness	0.757		
	Task coping	0.663		
	Surface approach	−0.585		
Factor 2	Neuroticism	0.854	14	Emotional
	Emotional coping	0.851		
Factor 3	Intrinsic motivation	0.843	11	Self-determined
	Motivation to care	0.834		
Factor 4	Agreeableness	0.836	8	Sociable
	Extraversion	0.515		
	Empathy	0.397		
Factor 5	Aptitude for medical studies	0.688	7	Intellectually flexible
	Openness	0.672		
Factor 6	Avoidant coping	0.716	7	Externally driven
	Extrinsic motivation	0.613		

### Differences in personal profiles and impact on selection

Selected students overall significantly differed from non-selected students by featuring a more diligent profile (Table 4). No interaction effect was found between selection and gender. Women's profiles differed from men's by being more emotional and sociable. Among selected students, the repeaters’ profile was similar to that of freshmen. However, at univariate levels, freshmen who passed ‘in two goes’ differed from those who passed ‘in one go’ on two facets: they were more emotional (mean [95% CI]: 0.11 [−0.11/0.33] vs. −0.30, [−0.61/−0.11]; *p*≤0.05) and less intellectually flexible (mean [95% CI]: −0.03 [−0.19/0.26], vs. 0.50 [0.20/0.89]; *p*≤0.05).

**Table 4:** Profile of selected and non-selected students, and of males and females

Facets	Selected	Not selected	*p* selected	Men	Women	*p* gender	*p* selected×gender
Overall			0.050*			0.001*	0.408
Diligent	0.20 [0.05; 0.35]	−0.11 [−0.33; 0.10]	0.010*	−0.05 [−0.24; 0.13]	0.17 [0.12; 0.32]	0.097	0.712
Emotional	−0.06 [−0.23; 0.11]	0.25 [0.05; 0.45]	0.185	−0.49 [0.68; −0.30]	0.40 [0.26; 0.55]	0.001*	0.908
Self-determined	−0.01 [−0.17; 0.16]	−0.01 [−0.22; 0.19]	0.887	−0.10 [−0.32; 0.11]	0.03 [−0.12; 0.19]	0.304	0.818
Sociable	−0.08 [−0.24; 0.09]	0.03 [−0.17; 0.24]	0.847	−0.34 [−0.55; −0.14]	0.11 [−0.05; 0.27]	0.001*	0.747
Intellectually flexible	0.18 [0.20; 0.34]	−0.10 [−0.29; 0.10]	0.113	0.24 [0.10; 0.46]	−0.06 [−0.21; 0.90]	0.107	0.565
Externally driven	−0.10 [−0.26; 0.07]	0.02 [−0.19; 0.24]	0.172	−0.20 [−0.42; 0.20]	0.06 [−0.09; 0.21]	0.361	0.018*

Mean [lower; upper limit of 95% confidence interval] of the regression variables obtained from principal component analysis.

* Multivariate ANOVA, *p*<0.05.

Logistic regression analysis (Table 5) confirmed that the profile's facet of ‘diligence’ increased the odds of being selected. In addition, it suggested that the facet ‘intellectually flexible’ also contributed to being selected and showed that repeaters got a double chance of being selected.

**Table 5:** Logistic regression analysis of students’ odds of being selected

Profile facets	OR [LL; UL 95% CI]	*p*
Repeater	2.2 [1.1; 4.4]	0.029
Gender (male)	1.6 [0.8; 3.1]	0.178
Diligent	1.4 [1.1; 1.9]	0.016
Intellectually flexible	1.4 [1.0; 1.8]	0.035
Self-determined	1.0 [0.8; 1.4]	0.770
Externally driven	0.9 [0.7; 1.2]	0.400
Sociable	0.9 [0.7; 1.2]	0.520
Emotional	0.8 [0.6; 1.2]	0.299

OR, odds ratio; LL, lower limit; UL, upper limit; CI, confidence interval.

## Discussion

In this study, the authors aimed to investigate whether medical students selected through a knowledge-based exam only had a different personal profile than non-selected students. The personal profile of first year medical students drafted from 15 measures and reduced by a PCA, highlighted six main facets, namely being diligent, emotional, self-determined, sociable, intellectually flexible, and externally driven. The main results suggested that students demonstrating higher diligence were more frequently selected. On the contrary, there were no relevant differences between selected and non-selected students according to personal characteristics such as motivation, empathy, or sociability, which are important to future caring doctors.

### Medical students’ personal profiles

The first facet ‘diligent’ consisted of being conscientious, adopting a deep learning approach, and coping with stress by targeting the task to be accomplished, in order to succeed. To our knowledge, the association between conscientiousness, learning approaches, and task coping, revealed in this study, has not been shown before. It therefore expands previous studies showing that high conscientiousness, allowing good organization, planning, and achievement orientation leads to higher use of concrete, well-adjusted strategies to face exam stress efficiently (18), and correlates positively with a deep-rooted approach to learning and negatively with a surface-rooted approach to learning (33, 34).

The profile's second facet, ‘emotional’, was characterized by neuroticism and emotional coping. These two characteristics are linked to the way students handle their emotions, and their association has been previously reported (18, 35). The particular context of uncertainty in being selected for medical studies through a competitive procedure in our institution might underscore this facet, by increasing negative feelings of anxiety and distress, toward a perceived highly threatening and unsolvable barrier to becoming a medical student. This vulnerability could lead students to employ emotional coping in order to reduce such a potential negative effect. The importance of this facet in our population could also be due to the high proportion of women, notably more emotional than their male counterparts.

The profile's third facet revealed in this study depicted ‘self-determined’ motivations. According to the self-determination theory, the association between intrinsic and care motivations in this profile might identify autonomous motivations generated internally by individuals displaying a genuine personal interest in others (23). Examples of autonomous motivation goals can be characterized by a marked sense of community involvement, personal growth, or society affiliations. Furthermore, high autonomous motivation seems to stimulate learning during medical studies (36, 37). Autonomous motivation is therefore an asset to any intensive preparation to face exams in a highly competitive context such as the selection to medical studies.

The fourth facet, ‘sociable’, was mostly characterized by a shared variance of extraversion and agreeableness. These two personality traits constitute the core of interpersonal relationships, and are therefore considered important for medical students’ future professional practice (38). This facet is particularly represented in the female population.

The fifth facet, ‘intellectually flexible’, combined aptitude for medical studies and openness to experience. The first core dimension of the openness trait is the intellect, that is, being quick in learning and highly insightful (39). Its shared variance with cognitive abilities is supported by the findings of personality studies (40).

The last facet, ‘externally driven’, was characteristic of an extrinsic motivation which in turn was associated with a preferred avoidant coping strategy. Extrinsic motivation is controlled and generated externally from outside pressure and/or rewards (23). Its association with avoidant coping suggests that being driven by external motives leads to easier and more frequent disengagement in studies, confirming previous findings in the field (41).

### Differences in personal profiles and impact on selection

We found the facet ‘diligent’ to be particularly developed in selected students and could show that it impacted positively the chance of being selected. High conscientiousness is the personality trait most frequently associated with academic achievement both in studies assessing students in general (11, 12) and medical students in particular (10, 13, 42). By contrast, evidence on predicting academic success by learning strategies is less conclusive. While some studies have shown a positive impact of a deep-rooted approach and a negative effect of a surface-rooted approach on academic performance (43), other studies failed to show the same effects, concluding that approach to learning is a subset of personality that has no clear independent impact on performance (12).

The facet ‘intellectually flexible’, represented by ‘openness’ and ‘aptitude for medical studies’, did not differ between selected and not selected students. However, in logistic regressions, it seemed to significantly increase students’ odds of being selected. The evidence found in the literature showing that the trait of “personality openness” improves academic performance in medical school (38) supports this finding, as also results from another study at our institution showing a clear correlation between scores on the aptitude test for medical studies and academic performance of first-year medical students, thereby increasing their chance of being selected (20).

The other facets, ‘emotional’, ‘self-determined’, ‘sociable’, and ‘externally driven’, did not impact selection, either positively or negatively. While it can be considered as reassuring that our selection process did not under-select students presenting the sociability facet, it did however not favor them and the question persists on the importance of medical students’ personality traits that would be more valuable and suitable for patients’ care beyond academic excellence.

In our study, women were proportionally less selected for medical studies than men. A factor potentially contributing to women's under-selection was their more diverse background with few having a scientific high school diploma, as compared with their male peers; this might have lowered their level of understanding during lectures and consequently their performance in MCQ tests, which were mainly based on knowledge in science. Our results are in line with a number of previous studies (16, 18, 35, 44) showing that women's profiles differ from that of men, revealing a stronger emotional facet which might be a reason for under-selection. However, we found no interaction effect between selection and gender, and the OR of gender on selection, although of 1.6 in favor of male students, was not statistically significant. The relatively small number of students following stratification by sex might not have had sufficient power to achieve statistical relevance and disentangle the apparently inconsistent results.

An additional finding of our study was that among selected students, those who were selected ‘in one go’ (after the first year of competing for medical studies) did not present a different profile from those who repeated their first year and passed ‘in two goes’. However, the former differed from the latter in the first year of selection regarding two facets, being more intellectually flexible and less emotional. On the contrary, the probability of repeaters being selected in their second year of applying for medical studies was more than double when compared with freshmen. We might therefore speculate whether repeaters developed new strategies after failing the first time round, which eventually allowed them to pass on a second chance. If true, these findings might indicate that offering a second chance with additional time to failing students would allow some of them to better adapt and cope with the stringent context of a first selection year.

### Strengths and limitations

Strengths of this study are the systematic collection of data and the high adherence to the survey by students who thoroughly completed the questionnaires, resulting in only minimal data missing. However, the study also has a number of limitations. First, this is a single-center study with a restricted number of students, which could limit the generalizability of the results. Thus, replication with other cohorts of students in distinct contexts would be necessary to ensure its general application. Second, our data were derived from a cross-sectional design and we could not ensure the reproducibility of the reported results. However, as mentioned above, the study is part of a larger cohort study and we will be able to test this aspect upon completion of the longitudinal collection of data. Third, not all instruments have been thoroughly validated which could limit the validity of our results, especially with regard to the motivation for becoming a doctor. The French versions of the R2-SPQ and the JSE-S have been validated by our group showing psychometric properties comparable with the original English versions and are awaiting publication. Finally, the use of self-reported answers to the questionnaires’ items did not provide an objective measure of the parameters under study, but on the contrary it ensured comparability with the evidence generated by studies using similar methodologies.

In conclusion, findings of this study show that for medical studies, a pure knowledge-based exam selects diligent, hardworking students with valuable traits, such as conscientiousness allied to a deep learning approach and to a task-focused coping style. In a recent report, Lievens defines conscientiousness as a ‘getting ahead’ skill that would be important during undergraduate preclinical years, whereas traits of extraversion, openness, and agreeableness were defined as ‘getting along’ skills which would determine success and professionalism in undergraduate clinical training and practice years (38). An ideal selection process should address both dimensions. In our specific context, the knowledge-based selection appears to take into account mainly the ‘getting ahead’ skills: this involves the risk of overlooking the ‘getting along’ competencies which would be required from students to become caring doctors, such as sociability, flexibility, empathy, openness, and agreeability (15, 45, 46). Our findings suggest that knowledge-based exams neither advantage nor preclude candidates with a more humane profile from being considered. The observation that women, who are particularly sociable, are under-selected by our process is however a matter of concern and needs to be further investigated.

Our results might contribute to further debate on the cost–benefits of the selection procedure to medical schools, by providing additional data on the profile of students selected through a knowledge-based exam only. Hence, it could be important for medical schools adopting this approach to complete their procedure in order to ensure that they select students able to become not only diligent but also caring doctors.

## References

[1] Royal College of Physicians and Surgeons of Canada (2012). http://www.royalcollege.ca/public/resources/aboutcanmeds.

[2] AAMC (2011). Behavioral and social science foundations for future physicians.

[3] AAMC-HHMI (2009). Scientific foundations for future physicians.

[4] Frank JR, Danoff D (2007). The CanMEDS initiative: implementing an outcomes-based framework of physician competencies. Med Teach.

[5] Koenig TW, Parrish SK, Terregino CA, Williams JP, Dunleavy DM, Volsch JM (2013). Core personal competencies important to entering students’ success in medical school: what are they and how could they be assessed early in the admission process?. Acad Med.

[6] Mahon KE, Henderson MK, Kirch DG (2013). Selecting tomorrow's physicians: the key to the future health care workforce. Acad Med.

[7] Siu E, Reiter HI (2009). Overview: what's worked and what hasn't as a guide towards predictive admissions tool development. Adv Health Sci Educ.

[8] Sawyer R (2013). Beyond correlations: usefulness of high school GPA and test scores in making college admissions decisions. Appl Meas Educ.

[9] McManus I, Woolf K, Dacre J, Paice E, Dewberry C (2013). The academic backbone: longitudinal continuities in educational achievement from secondary school and medical school to MRCP(UK) and the specialist register in UK medical students and doctors. BMC Med.

[10] Ferguson E, James D, Madeley L (2002). Factors associated with success in medical school: systematic review of the literature. BMJ.

[11] Poropat AE (2009). A meta-analysis of the five-factor model of personality and academic performance. Psychol Bull.

[12] Richardson M, Abraham C, Bond R (2012). Psychological correlates of university students’ academic performance: a systematic review and meta-analysis. Psychol Bull.

[13] Doherty EM, Nugent E (2011). Personality factors and medical training: a review of the literature. Med Educ.

[14] Monroe A, Quinn E, Samuelson W, Dunleavy DM, Dowd KW (2013). An overview of the medical school admission process and use of applicant data in decision making: what has changed since the 1980s?. Acad Med.

[15] Powis D (2015). Selecting medical students: an unresolved challenge. Med Teach.

[16] Costa PT, Terracciano A, McCrae RR (2001). Gender differences in personality traits across cultures: robust and surprising findings. J Pers Soc Psychol.

[17] Hojat M, Gonnella JS, Mangione S, Nasca TJ, Veloski JJ, Erdmann JB (2002). Empathy in medical students as related to academic performance, clinical competence and gender. Med Educ.

[18] Carver CS, Connor-Smith J (2010). Personality and coping. Annu Rev Psychol.

[19] Hänsgen KD, Spicher B (2011). EMS Eignungstest für das medizinstudium in der Schweiz.

[20] Cerutti B, Bernheim L, van Gessel E (2013). The predictive validity of the aptitude test for the performance of students starting a medical curriculum. Swiss Med Wkly.

[21] ILO (2012). International standard classification of occupations: ISCO-08.

[22] UNESCO (2011). International standard classification of education.

[23] Vansteenkiste M, Lens W, Deci EL (2006). Intrinsic versus extrinsic goal contents in self-determination theory: another look at the quality of academic motivation. Educ Psychol.

[24] Biggs JB, Kember D, Leung DYP (2001). The revised two factor study process questionnaire: R-SPQ-2F. Br J Educ Psychol.

[25] Socha A, Sigler EA (2014). Exploring and ‘reconciling’ the factor structure for the revised two-factor study process questionnaire. Learn Individ Differ.

[26] Endler N, Parker J (1998). Inventaire de coping pour situations stressantes – manuel.

[27] Costa PT, McCrae RR (1992). Revised NEO personality inventory (NEO-PI-R) professional manual, adaptation française.

[28] Aluja A, García O, Rossier J, García LF (2005). Comparison of the NEO-FFI, the NEO-FFI-R and an alternative short version of the NEO-PI-R (NEO-60) in Swiss and Spanish samples. Pers Individ Dif.

[29] Hojat M, Mangione S, Nasca TJ, Cohen MJM, Gonnella JS, Erdmann JB (2001). The Jefferson scale of physician empathy: development and preliminary psychometric data. Educ Psychol Meas.

[30] Tavakol S, Dennick R, Tavakol M (2011). Psychometric properties and confirmatory factor analysis of the Jefferson Scale of Physician Empathy. BMC Med Edu.

[31] Hojat M, Gonnella JS, Nasca TJ, Mangione S, Veloksi JJ, Magee M (2002). The Jefferson Scale of Physician Empathy: further psychometric data and differences by gender and specialty at item level. Acad Med.

[32] Lelorain S, Sultan S, Zenasni F, Catu-Pinault A, Jaury P, Boujut E (2013). Empathic concern and professional characteristics associated with clinical empathy in French general practitioners. Eur J Gen Pract.

[33] Duff A, Boyle E, Dunleavy K, Ferguson J (2004). The relationship between personality, approach to learning and academic performance. Pers Indiv Diff.

[34] Zhang LF (2003). Does the big five predict learning approaches?. Pers Indiv Diff.

[35] Connor-Smith JK, Flachsbart C (2007). Relations between personality and coping: a meta-analysis. J Pers Soc Psychol.

[36] Sobral DT (2004). What kind of motivation drives medical students’ learning quests?. Med Educ.

[37] Kusurkar RA, Ten Cate TJ, Vos CM, Westers P, Croiset G (2013). How motivation affects academic performance: a structural equation modelling analysis. Adv Health Sci Educ Theory Pract.

[38] Lievens F, Ones DS, Dilchert S (2009). Personality scale validities increase throughout medical school. J Appl Psychol.

[39] Caspi A, Roberts BW, Shiner RL (2005). Personality development: stability and change. Annu Rev Psychol.

[40] O'Connor MC, Paunonen SV (2007). Big five personality predictors of post-secondary academic performance. Pers Indiv Diff.

[41] Vansteenkiste M, Sierens E, Soenens B, Luyckx K, Lens W (2009). Motivational profiles from a self-determination perspective: the quality of motivation matters. J Educ Psychol.

[42] Lievens F, Coetsier P, De Fruyt F, De Maeseneer J (2002). Medical students’ personality characteristics and academic performance: a five-factor model perspective. Med Educ.

[43] Chamorro-Premuzic T, Furnham A (2008). Personality, intelligence and approaches to learning as predictors of academic performance. Pers Indiv Diff.

[44] Rantanen J, MetsÄPelto R-L, Feldt T, Pulkkinen LEA, Kokko K (2007). Long-term stability in the Big Five personality traits in adulthood. Scand J Psychol.

[45] Doherty EM, Kieran W (2013). Personality and medical education. Oxford textbook of medical education.

[46] Hojat M (2014). Assessments of empathy in medical school admissions: what additional evidence is needed?. Int J Med Educ.

